# Mapping Extracellular pH of Gliomas in Presence of Superparamagnetic Nanoparticles: Towards Imaging the Distribution of Drug-Containing Nanoparticles and Their Curative Effect on the Tumor Microenvironment

**DOI:** 10.1155/2017/3849373

**Published:** 2017-11-22

**Authors:** Samuel Maritim, Daniel Coman, Yuegao Huang, Jyotsna U. Rao, John J. Walsh, Fahmeed Hyder

**Affiliations:** ^1^Magnetic Resonance Research Center, Yale University, New Haven, CT, USA; ^2^Department of Biomedical Engineering, Yale University, New Haven, CT, USA; ^3^Department of Radiology and Biomedical Imaging, Yale University, New Haven, CT, USA

## Abstract

Since brain's microvasculature is compromised in gliomas, intravenous injection of tumor-targeting nanoparticles containing drugs (D-NPs) and superparamagnetic iron oxide (SPIO-NPs) can deliver high payloads of drugs while allowing MRI to track drug distribution. However, therapeutic effect of D-NPs remains poorly investigated because superparamagnetic fields generated by SPIO-NPs perturb conventional MRI readouts. Because extracellular pH (pH_e_) is a tumor hallmark, mapping pH_e_ is critical. Brain pH_e_ is measured by biosensor imaging of redundant deviation in shifts (BIRDS) with lanthanide agents, by detecting paramagnetically shifted resonances of nonexchangeable protons on the agent. To test the hypothesis that BIRDS-based pH_e_ readout remains uncompromised by presence of SPIO-NPs, we mapped pH_e_ in glioma-bearing rats before and after SPIO-NPs infusion. While SPIO-NPs accumulation in the tumor enhanced MRI contrast, the pH_e_ inside and outside the MRI-defined tumor boundary remained unchanged after SPIO-NPs infusion, regardless of the tumor type (9L versus RG2) or agent injection method (renal ligation versus coinfusion with probenecid). These results demonstrate that we can simultaneously and noninvasively image the specific location and the healing efficacy of D-NPs, where MRI contrast from SPIO-NPs can track their distribution and BIRDS-based pH_e_ can map their therapeutic impact.

## 1. Introduction

Treatment and management of glioblastoma, the most common and malignant form of primary brain tumors, represent an unmet clinical challenge [1]. While gliomas are relatively rare compared to other forms of cancer malignancies [1], they are characterized by the worst prognosis, with a 5-year survival of less than 10% [2]. Treatments fail because gliomas are highly invasive, the blood brain barrier (BBB) prevents drugs from reaching the tumor at therapeutic doses, and systemic toxicity limits benefits from therapy [3–5]. In addition, there is a lack of reliable in vivo methods that can simultaneously and noninvasively measure the delivery and therapeutic benefits of cancer drugs. Therapy can be greatly improved by delivering high drug doses specifically to the tumor (while minimizing systemic toxicities) and by timely and quantitative monitoring of the delivery and efficacy of these drugs.

The transport and delivery of therapeutic agents into the brain parenchyma are impeded by a dense network of capillary endothelial cells, pericytes, and perivascular macrophages, which together form the BBB [6]. In the healthy brain, the BBB allows a highly selective transport of endogenous substances (e.g., nutrients) that are critical to brain function while keeping out potentially harmful toxins and drugs that are circulating in the blood [7, 8]. However, the BBB is disrupted in several pathologies including high-grade gliomas leading to increased leakiness (i.e., hyperpermeability) [9, 10]. Breakthroughs in glioma imaging and chemotherapy exploit the fact that nanoparticles (NPs) loaded with drugs (D-NPs) and MRI contrast agents like superparamagnetic iron oxide (SPIO-NPs) can extravasate from the blood through the large vascular fenestrations into the tumor [11, 12]. The combination of increased vascular permeability and poor lymphatic clearance in tumors leads to accumulation of NPs in tumors through enhanced permeation and retention (EPR) [13]. While tumors, including gliomas, generally possess larger vascular fenestrations (and hence higher permeability) compared to healthy tissue, these fenestrations are highly dependent on the location of the vessels in the tumor (i.e., fewer abnormalities in vessels on tumor periphery and higher in the tumor core), and moreover the fenestrations depend on the age/size of the tumor (i.e., larger tumors tend to have more abnormalities) [14, 15]. The pores on tumor vasculature include caveolae, vesiculo-vacuolar organelles, and fenestrations that are on the order of 10–20 nm along with larger but sporadic interendothelial cell gaps, which are significantly larger than 200 nm in diameter [16–20]. Thus NPs like the Molday ION (or SPIO-NPs; 30–50 nm hydrodynamic diameter) can extravasate passively across the BBB of the tumor niche more effectively compared to the normal neuropil. Therefore extravasation and accumulation of NPs will vary between the tumor core, tumor boundary, and healthy tissue [21–25].

Tumor-specific delivery of D-NPs can be further enhanced by coating the D-NPs with ligands that target overexpressed receptors and/or transporters in tumors [26–29]. Despite these advances in targeting of D-NPs for delivering high drug payloads to tumors, the effect of these D-NPs on the tumor microenvironment remains largely unknown. SPIO-NPs have been evaluated and approved for clinical use as MRI contrast agents [30–37]. Because the MRI contrast generated by SPIO-NPs persists for a long time, SPIO-NPs have recently been combined with D-NPs and used to simultaneously image drug delivery and biodistribution with MRI [12, 38–41]. However, the large superparamagnetic fields generated by SPIO-NPs disturb most MRI molecular readouts.

Because low extracellular pH (pH_e_) is a hallmark of cancer pathogenesis and promotes tumor invasion and resistance to therapy [42–48], there is need for advanced pH_e_ mapping methods to enable monitoring of glioma invasion. Since some drugs only work in certain pH ranges, precise knowledge of pH_e_ can aid in choosing and tailoring therapeutic regimens [49–51]. Additionally, their therapeutic efficacy may be assessed by measuring their ability to raise and normalize pH_e_, for example, by drugs that alter pH_e_ directly or affect tumor's aerobic glycolysis. Many MRI methods exist for measuring and mapping pH_e_. Relaxation-based methods (e.g., with Gd^3+^) are highly dependent on the degree of tissue perfusion and local agent concentration thus making quantification of pH_e_ difficult [52]. pH_e_-sensitive MRI methods based on proton exchange (i.e., between water protons and protons of amide/amine and hydroxyl moieties) such as chemical exchange saturation transfer (CEST) are also dependent on agent concentration and may additionally be complicated by magnetization transfer effects [53]. Spectroscopic methods, for example, ^31^P MRS with 3-aminopropyl phosphonate (3-APP), which have pH_e_-sensitive exchangeable protons [54, 55], suffer from low spatial resolution and significant line broadening in the presence of SPIO-NPs [56].

We previously obtained pH_e_ maps in glioma-bearing rats with biosensor imaging of redundant deviation in shifts (BIRDS) using lanthanide agents, for example, thulium 1,4,7,10-tetraazacyclododecane-1,4,7,10-tetrakis(methylene phosphonate), TmDOTP^5−^ [57, 58]. Since the BIRDS platform is based on direct detection of the paramagnetically shifted resonances of the nonexchangeable protons on the agents (rather than their peak amplitude or effect on water relaxation rate), the pH_e_ readout with BIRDS is independent of agent concentration [59, 60]. The functional part of the pH sensitivity stems from the pH-sensitive exchangeable protons of the phosphonate groups on the agents. With advanced k-space sampling of ultra-fast chemical shift imaging (CSI), the spatiotemporal resolution of BIRDS has improved [61]. Previously we observed in vitro that the pH sensitivities and readout with BIRDS agents are not compromised by the presence of SPIO-NPs [62]. Here we hypothesized that BIRDS-based pH_e_ readout in glioma-bearing rats remains uncompromised by the presence of SPIO-NPs. We compared pH_e_ measured with TmDOTP^5−^ by BIRDS before and after infusion of SPIO-NPs in rats bearing 9L gliosarcomas and RG2 gliomas. We used different agent administration methods (renal ligation versus coinfusion with probenecid) to inhibit the rapid clearance of the agent by the renal system. In addition, we compared the transverse relaxation rate enhancement from SPIO-NPs across brain regions. Our results suggest that we can use the MRI contrast from SPIO-NPs to track the distribution of D-NPs and then use the BIRDS-based pH_e_ readout to map their therapeutic impact.

## 2. Materials and Methods

TmDOTP^5−^ for BIRDS was purchased from Macrocyclics Inc. (Plano, TX, USA), while SPIO-NPs (Molday ION) were purchased from BioPAL Inc. (Worcester, MA, USA). The Molday ION (10 mg Fe/mL, dextran-coated, hydrodynamic diameter 30 nm, zeta potential −4.8 mV) were used without further modification or dilution to avoid altering their physical properties. Probenecid (used for temporary inhibition of renal clearance) was purchased from Sigma-Aldrich (St. Louis, MO, USA). Fischer 344 rats (male, 200–250 g) were obtained from Yale University vendors. RG2 and 9L tumor cell lines were purchased from American Type Culture Collections (Manassas, VA, USA). All animal experiments were conducted in accordance with Yale University's approved institutional animal care and use committee (IACUC) protocols. Tumor inoculation, animal preparation, and handling were conducted as described in our previous work [57, 58]. In vivo magnetic resonance (MR) scans were conducted on a 9.4T Agilent (Santa Clara, CA, USA) or Bruker (Billerica, MA, USA) horizontal-bore spectrometer with a 1.4-cm ^1^H surface RF coil.

### 2.1. Tumor Inoculation

The RG2 and 9L tumor cell lines were cultured and grown at 37°C and 5% CO_2_ in DMEM media containing 10% heat-activated fetal bovine serum and 1% penicillin-streptomycin. The cells were harvested when they reached 80% confluence and suspended in serum-free media for inoculation. Rats were anesthetized with 3% isoflurane and placed on a stereotactic holder. A heating pad was used to maintain the rat at physiological temperature (36-37°C). An aliquot volume of 5 *μ*L with RG2 cells (1,250 cells) or 9L cells (100,000 cells) was injected into the right striatum 3 mm laterally to the right of bregma and 3 mm below the dura using a 10 *μ*L Hamilton syringe fitted with a 26-gauge beveled needle. The 5 *μ*L volume was injected over the course of 5 minutes and the needle was left in place for an additional 5 minutes after the infusion stopped. The needle was then withdrawn slowly to prevent backflow of the cells. The cranial burr hole was sealed with bone wax. The scalp was sutured and treated with antibiotics to prevent infection. Meloxicam (1 mg/kg) was administered to prevent pain and inflammation.

### 2.2. Animal Preparation and Scanning

The tumor-bearing rats were scanned ~3 weeks after tumor inoculation when the tumor diameter was at least ~3 mm. The rats were anesthetized with 2% isoflurane, tracheotomized, and artificially ventilated (70% N_2_O/30% O_2_). The rats were placed on a heating pad to keep them warm during surgery. A femoral vein was cannulated with a PE-10 line for contrast agent administration (1 mmol/kg for TmDOTP^5−^ and 14 mg Fe/kg for SPIO-NPs). A femoral artery was cannulated with a PE-50 line for monitoring animal physiology (pCO_2_, pO_2_, pH, blood pressure) throughout the experiment. The rat was then anesthetized with *α*-chloralose using an intraperitoneal line. To inhibit renal clearance and enhance contrast agent extravasation into the extracellular space and accumulation in the tumor, rats either received a coinfusion of TmDOTP^5−^ and probenecid (*n* = 5) or underwent renal ligation and infusion of TmDOTP^5−^ alone (*n* = 3). While renal ligation inhibits clearance efficiently, it is not suitable for longitudinal studies. Previously, we demonstrated that probenecid temporarily inhibited renal clearance when coinjected with the agent, thus enabling longitudinal studies and obviating the need for invasive renal surgeries [58]. Probenecid (100 mg/kg) was infused for 10 minutes (24.5 *μ*L/min), followed by a waiting period of 20 minutes, and then coinfused slowly with TmDOTP^5−^ over a period of 90 minutes. A water-heating blanket was used to maintain body temperature of the animals between 36 and 37°C over the course of the experiment. A rectally placed fiber optic probe was used to monitor the body temperature during the scans.

### 2.3. MRI and BIRDS

In vivo transverse relaxation rate (*R*_2_) maps were obtained using a standard spin-echo sequence with 11 slices, 128 × 128 in-plane resolution, 1 mm slice thickness, field of view (FOV) 25 × 25 mm^2^, recycle time (TR) 6 s, and 12 different values of echo time (TE) from 10–120 ms. The transverse relaxivity (*r*_2_) of Molday ION (SPIO-NPs) was measured in vitro using the same pulse sequence using samples of varying concentrations of Molday ION (1 mg/kg to 15 mg/kg). The relaxivity was calculated from the slope of the linear fit of *R*_2_ versus concentration. Although extreme pH changes can significantly alter properties of NPs, Liu et al. showed that the zeta potentials and hydrodynamic diameters of dextran-coated SPIO-NPs are fairly stable at physiologically relevant pH and ionic concentrations [63]. They showed that, between pH 4–8 and media of different ionic strength (0–140 mM), there was no aggregation of SPIO-NPs and that the change in hydrodynamic diameter of SPIO-NPs was less than 10 nm, while the change in zeta potential was less than 10%. Nevertheless, extreme pH changes could affect the physical features of SPIO-NPs. For example, a pH less than 4 could degrade the SPIO-NPs altogether, while a pH greater than 10 could lead to significant aggregation. Because we did not modify the SPIO-NPs or change their media and pH, we do not expect property changes within the pH_e_ range of tumors, normal tissue, and blood.

The rats were infused with TmDOTP^5−^ and the 3D CSI acquisition was started 40 minutes after TmDOTP^5−^ infusion in both the probenecid coinfused and renal-ligated rats. TmDOTP^5−^ and similar lanthanide agents have previously been shown to cross the BBB and have been used to map whole brain pH_e_ and temperature by BIRDS in healthy rodents [60, 64, 65]. We previously proposed that these agents slowly diffuse in the brain through the fenestrated vessels of circumventricular organs [66, 67]. Moreover, diffusion of these agents from blood vessels into the extracellular space is enhanced by the high concentration gradient achieved by inhibition of renal clearance using renal ligation or coinfusion of the agent with probenecid [58, 60, 64].

The 3D CSI datasets were acquired with a reduced spherical encoding of k-space, as previously described [57], with a TR of 5 ms, FOV of 25 × 25 × 25 mm^3^, and a nominal voxel resolution of 1 *μ*L. A dual-banded refocused 90° Shinnar-Le Roux (SLR) pulse of 35 kHz bandwidth, 90 kHz separation, and 205 *μ*s duration was used to selectively excite the H2/H3 and H6 protons of TmDOTP^5−^ (i.e., on either side of water). The phase encoded gradient duration was 160 *μ*s, the spectral width was 250 kHz, and the acquisition time was 4.1 ms. The total acquisition time for each 3D CSI dataset scan was 12 minutes. First a pH_e_ map was acquired before the SPIO-NPs injection. Then a spin-echo dataset was obtained to determine the *R*_2_ enhancement induced by TmDOTP^5−^. Next, the TmDOTP^5−^ infusion was stopped and SPIO-NPs were injected slowly (over 5 minutes). Then another spin-echo dataset was obtained 15 minutes after the infusion of SPIO-NPs to determine the additional *R*_2_ enhancement due to SPIO-NPs. Finally, infusion of remaining TmDOTP^5−^ dose was then resumed and another pH_e_ map was obtained after infusion of SPIO-NPs.

*R*_2_ maps were obtained by fitting the absolute MRI intensity at different TEs to a single exponential function using Matlab (Mathworks Inc., Natick, MA, USA). *R*_2_ values from the 3 conditions (i.e., no contrast agent, after TmDOTP^5−^ infusion, and after SPIO-NPs infusion) were compared to determine the relaxation enhancement of each contrast agent. Average *R*_2_ values were measured in regions of interest (ROIs), where 1 mm circular rings were taken from the center of mass of the tumor. The tumor edge was defined as regions 1 mm immediately outside the MRI-defined tumor core. Comparing the measured *R*_2_ against the relaxivity of Molday ION allowed the amount of SPIO-NPs in each region to be approximated.

The 3D CSI datasets were used to create maps of the H2, H3, and H6 resonances of TmDOTP^5−^ before and after infusion of SPIO-NPs. The linewidth (LW) of the H6 resonance was measured to generate LW maps and create histograms before and after infusion of SPIO-NPs. While any of the three resonances could have been used to make the LW maps, H6 was chosen because it had the highest signal-to-noise ratio (SNR). BIRDS-based pH_e_ maps of the brain obtained with TmDOTP^5−^ were calculated as previously described [57, 58, 60, 62]. Briefly, the 3D CSI datasets were reconstructed to a 25 × 25 × 25 matrix using an in-house Matlab script. pH_e_ was calculated by fitting the H2, H3, and H6 resonances (i.e., *δ*_2_, *δ*_3_, and *δ*_6_, respectively) to

(1) $$\begin{array}{c} {pH}_{e}=a_{0}+\sum_{k=2,3,6}a_{1}^{k}\delta_{k}+\sum_{k=2,3,6}\sum_{j=2,3,6}a_{2}^{kj}\delta_{k}\delta_{j}, \end{array}$$

where the coefficients *a*_0_, *a*_1_^*k*^, and *a*_2_^*kj*^ were calculated from linear least-squares fit of pH_e_ as a function of the resonances *δ*_2_, *δ*_3_, and *δ*_6_ [60]. Average pH_e_ values before and after infusion of SPIO-NPs were determined as a function of distance from the center of mass of the tumor, similar to the procedure described above for the *R*_2_ maps.

### 2.4. Prussian Blue Iron Staining

Rats were sacrificed at the end of the experiments and brains were perfusion-fixed in 4% paraformaldehyde for Prussian blue iron staining to assess the distribution of SPIO-NPs. 10 *μ*m thick coronal sections of the fixed tissue were incubated in a solution of 4% potassium ferrocyanide and 4% hydrochloric acid twice for 10 minutes and then counterstained with nuclear fast red. Regions with Fe^3+^ (from SPIO-NPs) were expected to stain blue due to formation of ferric ferrocyanide.

## 3. Results

**Figure 1:**
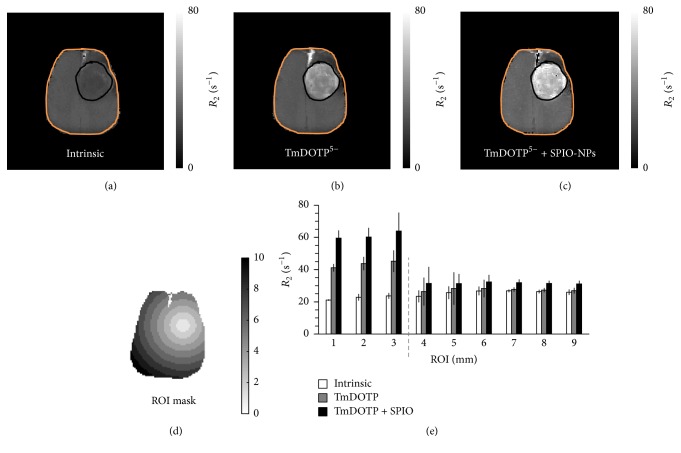
Transverse relaxation rate (*R*_2_) maps of an RG2 glioma-bearing rat that underwent renal ligation for TmDOTP^5−^ infusion, (a) without any contrast agent, (b) after infusion of TmDOTP^5−^, and (c) after infusion of SPIO-NPs. The scale bar in (a–c) denotes *R*_2_ values from 0 to 80 s^−1^. Compared to the *R*_2_ map before TmDOTP^5−^ infusion (a), the *R*_2_ enhancement was observed throughout the brain after TmDOTP^5−^ infusion (b), but superior *R*_2_ enhancement and tumor delineation were observed following infusion of SPIO-NPs which also had cumulative effects from infusion of TmDOTP^5−^ (c). The contrast enhancement from both TmDOTP^5−^ and TmDOTP^5−^ with SPIO-NPs was region-specific, with highest enhancement in the tumor core and limited enhancement outside the tumor (relative to the intrinsic contrast, (a)). The black outline in (a–c) denotes the tumor boundary, which is based on the superior MRI contrast after infusion of SPIO-NPs. The region of interest (ROI) mask based on 1 mm circular rings from the tumor center (d) was used to generate the radial *R*_2_ distribution histogram of these ROIs (e). Scale bar in (d) denotes 0 to 10 mm diameter circular ROIs (portrayed on a representative rat brain slice). The gray dashed line in (e) denotes the demarcation between tumor and nontumor regions. The amount of SPIO-NPs in the tumor was 4.3 times greater than in the healthy tissue suggesting a preferential extravasation and accumulation of SPIO-NPs in the tumor. See Figure S1 for examples of Prussian blue staining for SPIO-NPs of an RG2 glioma-bearing rat that underwent renal ligation for TmDOTP^5−^ infusion. See Figure S2 for examples of *R*_2_ maps of an RG2 glioma-bearing rat that underwent coinfusion of probenecid and TmDOTP^5−^.

The *R*_2_ maps before any contrast agent infusion (Figure 1(a)), after TmDOTP^5−^ infusion (Figure 1(b)), and after the infusion of SPIO-NPs (Figure 1(c)) are shown for a renal-ligated rat bearing an RG2 tumor. While tumor localization was obtained in all three MRI cases, much better delineation was observed upon enhancements by TmDOTP^5−^ alone or TmDOTP^5−^ + SPIO-NPs. While *R*_2_ increases were observed after infusion of TmDOTP^5−^ (relative to the intrinsic contrast), a superior MRI contrast was observed after the infusion of SPIO-NPs. The circular ROIs that were drawn from the tumor center are shown in Figure 1(d). The *R*_2_ relaxation enhancement was ROI-dependent with higher *R*_2_ values inside the tumor and lower *R*_2_ outside the tumor (Figure 1(e)). Since *R*_2_ enhancement was dependent on the concentration of the paramagnetic agents, the observed ROI-specific *R*_2_ enhancement suggests that the extravasation (and accumulation) of both TmDOTP^5−^ and SPIO-NPs was highest in the tumor core and lower in regions farthest from the tumor's center of mass.

The *R*_2_ values in the tumor (boundary marked by black outlines in Figures 1(a)–1(c)) were 22.5, 43.4, and 61.2 s^−1^ before contrast agent infusion, after infusion of TmDOTP^5−^, and after infusion of SPIO-NPs, respectively. For the healthy/nontumor tissue (contralateral side), the *R*_2_ values were 25.8, 27.3, and 31.5 s^−1^ before contrast agent administration, after infusion of TmDOTP^5−^, and after infusion of SPIO-NPs, respectively. The measured *R*_2_ relaxivity of Molday ION at 9.4 T in vitro was 2.45 s^−1 ^mg^−1^ Fe/kg. By comparing the *R*_2_ enhancement by SPIO-NPs against the relaxivity of the Molday ION, the average concentration of SPIO-NPs in the tumor (ROIs 1–3 mm) was determined to be 7.27 mg Fe/kg. In healthy/nontumor tissue (ROIs 4–9 mm), there was a 4.1 s^−1^ change in *R*_2_ with SPIO-NPs, which corresponds to 1.69 mg Fe/kg. Thus, the concentration of SPIO-NPs in the tumor was 4.3 times greater than in healthy/nontumor tissue, suggesting a fourfold enhanced extravasation/accumulation in the tumor.

Given the physical characteristics of Molday ION, we anticipate that the induced MRI effect is from SPIO-NPs within the extracellular milieu and calculating the concentration of SPIO-NPs in vivo should not be significantly affected by using the relaxivity measured in vitro. Girard et al. showed that the relaxivity of SPIO-NPs internalized in cells was lower than that of freely dispersed (in vitro) SPIO-NPs by as much as up to 4 times [68]. Taylor et al. also showed that the relaxivity of Molday ION internalized in cells was 4 times lower than the relaxivity in solution [69]. If we assume the relaxivity of Molday ION in vivo is 4 times lower than what was measured in vitro, then the calculated concentration of SPIO-NPs in both the tumor and healthy brain would be 4 times higher, but the relative distribution in tumor versus healthy/nontumor tissue would remain the same. For example, if we assume a 4x lower in vivo relaxivity (0.61 mg^−1^ s^−1^ in vivo versus 2.45 mg^−1^ s^−1^ in vitro), the concentration of SPIO-NPs in the tumor would be 29.16 mg Fe/kg while the concentration in the healthy tissue would be 6.72 mg Fe/kg (4.3 times lower than in the tumor). However, we expect that most of the SPIO-NPs will accumulate in the extracellular space where the microenvironment is more similar to the in vitro situation than that of SPIO-NPs internalized in cells. Moreover, we do not expect the relaxation of SPIO-NPs to change significantly over the pH range of our in vivo studies (i.e., pH_e_ 6.8 in tumors to 7.3 in healthy/nontumor tissue). Liu et al. and others have shown that the *R*_2_ of dextran-coated SPIO-NPs was not significantly different over this pH range [63, 70]. Using different concentrations of Molday ION, Shu et al. showed that the *R*_2_ increase with increasing Molday ION dose was uniform across different brain regions [71]. Thus we expect the relaxivity of SPIO-NPs calculated in vitro to be a good approximation of the in vivo situation. Although we expect our concentration estimation to be only minimally affected, nevertheless these values should be considered “apparent” concentrations.

The preferential distribution of SPIO-NPs in tumors over healthy/nontumor tissues was tested with Prussian blue staining for Fe^3+^. Although the results from Prussian blue staining are not quantitative, regions that showed higher levels of SPIO-NPs were stained blue, indicating presence of Fe^3+^ (see Figure S1 in the Supplementary Material available online at https://doi.org/10.1155/2017/3849373). The Prussian blue stained images show an abundance of SPIO-NPs in the tumor, but very little staining on the healthy/nontumor contralateral side of the brain, supporting the enhanced accumulation of SPIO-NPs in the tumors observed with *R*_2_ mapping.

Since the *R*_2_ data shown in Figure 1 is from a renal-ligated rat, we obtained similar data from a rat that underwent coinfusion of TmDOTP^5−^ and probenecid (Figure S2). The amount of SPIO-NPs in the tumor was 2 times higher than in nontumor tissue (i.e., 4.5 versus 2.2 mg Fe/kg). In this case, the *R*_2_ enhancement (Figure S2) was slightly lower than that observed in a renal-ligated rat (Figure 1), which is possibly due to higher TmDOTP^5−^/SPIO-NPs concentration buildup in a renal-ligated rat.

**Figure 2:**
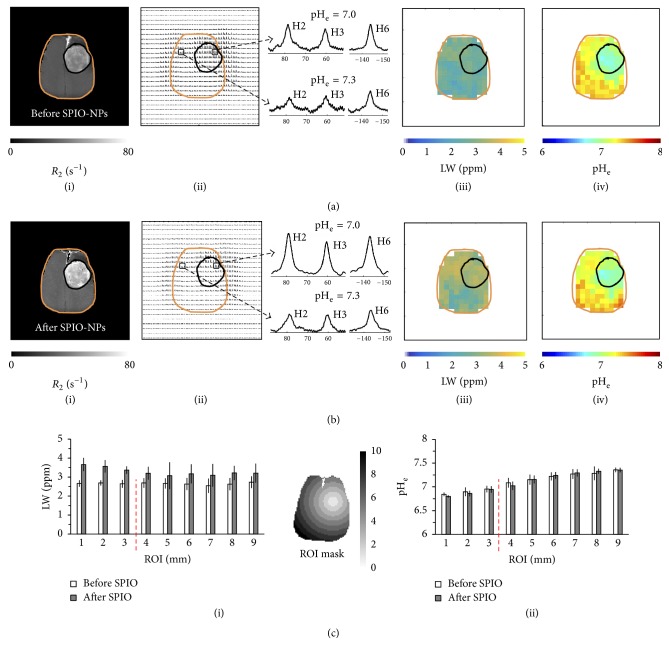
Multimodal data of relaxation rate (*R*_2_), chemical shift imaging (CSI), linewidth (LW), and extracellular pH (pH_e_) maps obtained for the same RG2 tumor-bearing rat in Figure 1, which had undergone renal ligation. ((a)(i)–(iv)) represent maps before the SPIO-NPs infusion while ((b)(i)–(iv)) represent the maps after the SPIO-NPs infusion. The *R*_2_ maps were used to delineate and localize the tumor (black outline) and brain (orange outline) boundaries on the CSI, LW, and pH_e_ maps. *R*_2_ values inside the tumor increased significantly after infusion of SPIO-NPs. The CSI maps were used to create the LW maps and pH_e_ maps. The LW increased after SPIO-NPs infusion especially in the tumor. The pH_e_ values within the tumor core and also on the tumor margin were lower than in the healthy/nontumor regions. The panels between the CSI and the LW maps show examples of ^1^H spectra of TmDOTP^5−^ protons from voxels inside and outside the tumor, revealing a significant intratumoral and peritumoral LW and pH_e_ difference. A more detailed comparison was done using a region of interest (ROI) analysis of LW ((c)(i)) and pH_e_ ((c)(ii)) maps before and after the infusion of SPIO-NPs, using the ROI mask shown. The scale bar in the mask denotes 0 to 10 mm diameter circular ROIs (portrayed on a representative rat brain slice). The red dashed line denotes the demarcation between tumor (ROIs 1–3) and tumor edge (ROI 4)/nontumor regions (ROIs 5–9). See Figure S3 for an example of multimodal data of an RG2 tumor rat that underwent coinfusion of probenecid and TmDOTP^5−^.

Because acidic pH_e_ is a hallmark of tumor pathology [72, 73], we obtained brain pH_e_ maps in glioma-bearing rat brains with BIRDS using TmDOTP^5−^ before and after infusion of SPIO-NPs. We previously demonstrated that high concentrations of SPIO-NPs increase the LW of the TmDOTP^5−^ proton resonances in vitro [62]. In the current work, in addition to pH_e_ maps, we also calculated the LW of the H6 proton of TmDOTP^5−^ in each voxel in the brain, before and after infusion of SPIO-NPs. Multimodal data (*R*_2_ maps (i), CSI maps (ii), LW maps (iii), and pH_e_ maps (iv)) before (Figure 2(a)) and after (Figure 2(b)) infusion of SPIO-NPs for the same RG2 tumor-bearing rat shown in Figure 1, which had undergone renal ligation, were obtained. The *R*_2_ maps (Figures 2(a)(i) and 2(b)(i)) were used to delineate and localize the tumor (black outline) and brain (orange outline) boundaries on the CSI, LW, and pH_e_ maps. The CSI maps (Figures 2(a)(ii) and 2(b)(ii)) were used to create the LW maps (Figures 2(a)(iii) and 2(b)(iii)) and pH_e_ maps (Figures 2(a)(iv) and 2(b)(iv)). Examples of ^1^H spectra of TmDOTP^5−^ protons from voxels inside and outside the tumor—illustrated in the panel between the CSI maps and LW maps—show that there is a significant intratumoral and peritumoral LW and pH_e_ differences. The SNR was higher in the spectra after infusion of SPIO-NPs than before because TmDOTP^5−^ infusion was resumed 15 minutes after the end of SPIO-NPs infusion. While the CSI maps (Figures 2(a)(ii) and 2(b)(ii)) show regionally varying TmDOTP^5−^ intensities, after infusion of SPIO-NPs, there is a clear variation in the LW maps, increasing from ~2.5 ppm globally before the infusion (Figure 2(a)(iii)) to ~3.7 ppm inside the tumor and ~3.4 ppm in the healthy/nontumor contralateral side of the brain (Figure 2(b)(iii)). A detailed ROI analysis of the average LWs shows similar LWs inside and outside the tumor before infusion of SPIO-NPs (Figure 2(c)(i), white bars). However, upon infusion of SPIO-NPs (Figure 2(c)(i), gray bars), the average LW increased (from 2.6 to 3.5 ppm) in the tumor (ROIs # 1–3) and to a lesser extent (from 2.6 to 3.1 ppm) outside the tumor (ROIs # 4–9). The LW broadening in the tumor correlated with the *R*_2_ enhancement suggesting that the LW broadening was due to higher concentration of SPIO-NPs in the tumor.

The pH_e_ maps (Figures 2(a)(iv) and 2(b)(iv)) were obtained by fitting the chemical shifts of the H2, H3, and H6 protons of TmDOTP^5−^ to equation (1) as previously described [60]. Although the CSI maps show regional variation of TmDOTP^5−^ proton intensities, both before and after infusion of SPIO-NPs (Figures 2(a)(ii) and 2(b)(ii)), the pH_e_ calculation depends only on the chemical shifts of the nonexchangeable TmDOTP^5−^ protons and is independent of their concentration (or peak intensity) [59, 60]. The pH_e_ maps of RG2 tumors show lower pH_e_ within the tumor core, but also beyond the tumor boundary, which is in good agreement with previous observations of this aggressive tumor type [57, 58]. Before injection of SPIO-NPs the average pH_e_ was 7.0 ± 0.1 within the tumor and 7.3 ± 0.1 in the healthy/nontumor tissue on the contralateral side for the RG2 tumor-bearing brain (Figure 2(a)(iv)). After injection of SPIO-NPs similar average pH_e_ values were observed in these regions (i.e., 7.0 ± 0.1 in the tumor and 7.3 ± 0.1 in healthy/nontumor tissue; Figure 2(b)(iv)). These in vivo results are consistent with our earlier in vitro report which showed that the pH readout and sensitivities of TmDOTP^5−^ are unaffected by the presence of paramagnetic agents like SPIO-NPs and Gd^3+^ agents [62]. Moreover, a detailed ROI analysis of the spatial pH_e_ distribution shows that the average pH_e_ increased as the ROI is positioned farther from the center of mass of the tumor (Figure 2(c)(ii)). However, no significant differences were observed between the average pH_e_ values in each ROI before and after the SPIO-NPs infusion, indicating that BIRDS-based pH_e_ mapping is not affected by the presence of SPIO-NPs.

Similar *R*_2_, CSI, LW, and pH_e_ maps as those shown in Figure 2 were obtained from rats, bearing RG2 tumors, that underwent coinfusion of TmDOTP^5−^ and probenecid (Figure S3). The results show that these distributions were similar to those observed in renal-ligated rats. Generally, the LWs increased after infusion of SPIO-NPs in all regions of the brain, but higher LW increases were observed inside the tumor. Before infusion of SPIO-NPs, the pH_e_ was 6.85 ± 0.03 in the tumor and the pH_e_ was 7.15 ± 0.06 in healthy/nontumor tissue (Figure S3 (A)(iv)). After infusion of SPIO-NPs, the pH_e_ was 6.86 ± 0.07 in the tumor and the pH_e_ was 7.17 ± 0.06 in healthy/nontumor tissue (Figure S3 (B)(iv)). The pH_e_ of the tumor edge (ROI 4) was also relatively acidified (pH 6.98 ± 0.13 before and 6.90 ± 0.09 after infusion of SPIO-NPs) compared to healthy/nontumor tissue farthest from the tumor core (ROIs 5–9).

**Figure 3:**
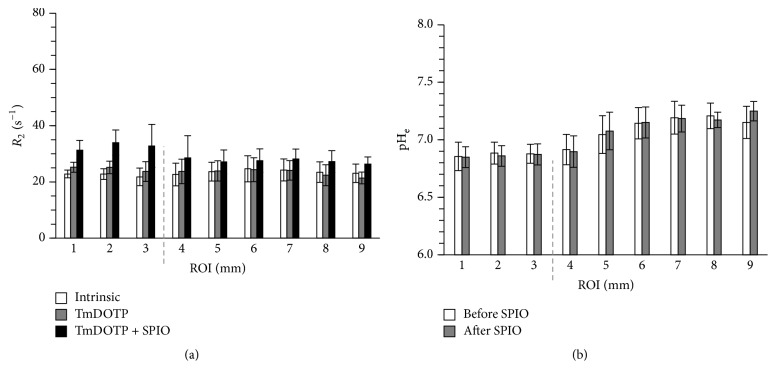
Region of interest (ROI) analysis for the average relaxation rate (*R*_2_) and extracellular pH (pH_e_) before and after infusion of SPIO-NPs for all RG2 tumor-bearing rats that underwent coinfusion of TmDOTP^5−^ and probenecid (*n* = 5). The ROI analysis is based on concentric 1 mm circular rings drawn from the center of mass of the tumor. (a) Average *R*_2_ values in different ROIs for intrinsic contrast, after infusion of TmDOTP^5−^, and after infusion of TmDOTP^5−^ with SPIO-NPs. The average *R*_2_ enhancement was highest inside the tumor (ROIs 1–3) compared to tumor edge (ROI 4) and nontumor regions (ROIs 5–9). The dashed line represents the tumor edge. Small *R*_2_ enhancement was observed after infusion of TmDOTP^5−^. However, much higher *R*_2_ enhancement was observed upon infusion of SPIO-NPs. See Table 1 for details. (b) The average pH_e_ values in different ROIs before and after infusion of SPIO-NPs. The pH_e_ values measured before and after infusion of SPIO-NPs were similar, both inside and outside the tumor. The pH_e_ was lowest in the tumor and highest in the healthy/nontumor tissue farthest from the tumor. Low pH_e_ was also measured on the tumor margin. See Table 2 for details.

Figure 3 shows the ROI analysis for *R*_2_ and pH_e_ before and after infusion of SPIO-NPs for all RG2 tumor-bearing rats that underwent coinfusion of TmDOTP^5−^ and probenecid (*n* = 5). The *R*_2_ enhancement was region-dependent (Figure 3(a); Table 1). Small *R*_2_ enhancement was observed after infusion of TmDOTP^5−^, where *R*_2_ increased by 2.2 s^−1^ in the tumor, 1.1 s^−1^ in the tumor edge, and no significant increase in the healthy/nontumor tissue. The tumor edge was defined as a circular ROI just 1 mm outside of the MRI defined tumor core. It is important to identify and analyze the tumor edge because after radiation therapy, it becomes edematous and harbors most therapy-resistant cells. Additionally, greater *R*_2_ enhancement was observed upon infusion of SPIO-NPs (i.e., *R*_2_ increase of 10.2 s^−1^ in tumor, 5.9 s^−1^ in tumor edge, and 4.1 s^−1^ in healthy/nontumor tissue). In contrast to the *R*_2_ measurements, the average pH_e_ values were not affected by the SPIO-NPs infusion (Figure 3(b); Table 2). However, pH_e_ varied across regions; pH_e_ was lowest (6.9 ± 0.1) in the tumor and highest (7.2 ± 0.1) in the healthy/nontumor tissue farthest from the tumor. Low pH_e_ (6.9 ± 0.1) was also measured on the tumor edge. While the pH_e_ of the tumor edge in RG2 gliomas was acidic relative to healthy/nontumor tissue, the *R*_2_ enhancement between the tumor edge and the healthy/nontumor tissue were similar, suggesting that the vasculature in the tumor margin was still intact despite the acidic transformation of their microenvironment. Future experiments should look at the vascularization inside, around, and far beyond the tumor boundary, for example, with dynamic contrast enhanced MRI and with epidermal growth factor receptor staining.

**Figure 4:**
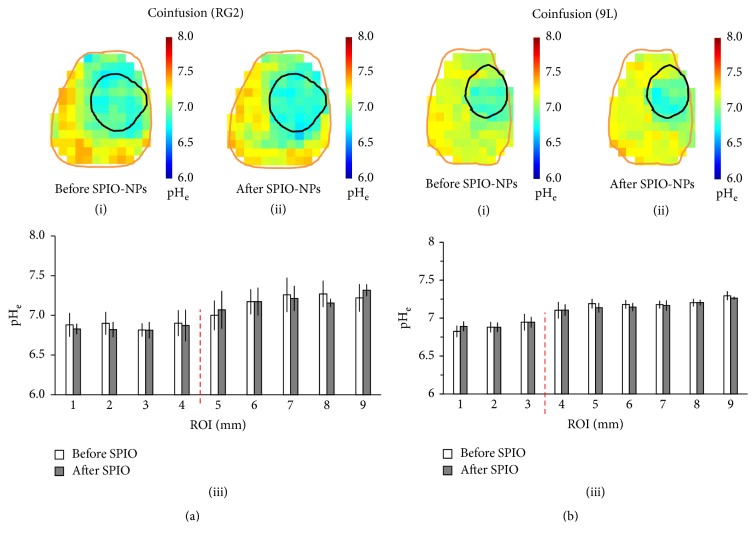
Comparison of pH_e_ maps for (a) RG2 glioma and (b) 9L gliosarcoma before and after infusion of SPIO-NPs in rats that underwent coinfusion of TmDOTP^5−^ and probenecid. In both (a) and (b), (i) and (ii) represent the pH_e_ maps before and after SPIO-NPs infusion, respectively, while (iii) depicts a detailed ROI analysis. See Figure 1 for details of the ROI mask. In (a), the pH_e_ inside the more aggressive RG2 glioma was typically lower than in the healthy/nontumor tissue, but diffuse acidification was observed well-beyond the MRI-defined tumor boundary. Thus the pH_e_ slowly increased as the distance from the tumor core increased. In (b), the pH_e_ inside the less aggressive 9L gliosarcoma was also lower than in the healthy/nontumor tissue, but the acidification did not extend beyond the MRI-defined tumor boundary, before and after infusion of SPIO-NPs. See Figure S4 for a comparison of regional pH_e_ dependence on the method used for inhibition of renal clearance (renal ligation versus probenecid).

In addition to measurements obtained in the aggressive RG2 glioma, we also acquired pH_e_ maps before and after infusion of SPIO-NPs in rats bearing the less aggressive 9L gliosarcoma (*n* = 4) using coinfusion of TmDOTP^5−^ and probenecid (Figure 4). The pH_e_ maps of the aggressive RG2 tumor (Figure 4(a)(i) before versus Figure 4(a)(ii) after infusion of SPIO-NPs) showed a lower pH_e_ within the tumor region, but the acidification was diffuse and occurred also beyond the MRI-defined tumor boundary (see also Figures 2 and S3). Previously, it was reported that the diffuse acidification of pH_e_ beyond the RG2 tumor boundary correlated with increased expression of the proliferation marker Ki-67 [57]. In contrast, the pH_e_ maps of the less aggressive 9L gliosarcoma showed lower pH_e_ only within the MRI-defined tumor core (Figure 4(b)(i) before versus Figure 4(b)(ii) after SPIO-NPs). A detailed ROI analysis of the pH_e_ maps shows that, for the RG2, the pH_e_ slowly increases with the distance from the tumor core (Figure 4(a)(iii)), whereas for the 9L tumor the pH_e_ is highest outside the tumor boundary and is distance-independent (Figure 4(b)(iii)). Moreover, the regional pH_e_ trends observed with BIRDS were not dependent on the type of infusion method, that is, coinfusion of TmDOTP^5−^ and probenecid versus infusion of TmDOTP^5−^ after renal ligation (Figure S4).

## 4. Discussion

Elevated aerobic glycolysis in gliomas leads to elevated lactic acid and proton production, which upon extrusion from the intracellular compartment results in acidification of the extracellular milieu [44]. Additionally, because the BBB is disrupted in gliomas, NPs loaded with imaging agents (e.g., SPIO-NPs) selectively permeate into and accumulate within tumors. In the present study, a region-specific *R*_2_ enhancement from extravasation of SPIO-NPs was observed, with higher *R*_2_ increases inside the tumor and smaller *R*_2_ increases outside the tumor. Although SPIO-NPs affected MRI contrast in all tissues, excellent SPIO-induced MRI contrast delineated the glioma boundary due to greater extravasation of SPIO-NPs from the vasculature into the tumor relative to healthy/nontumor tissue. We also measured pH_e_ with BIRDS using TmDOTP^5−^ before and after infusion of SPIO-NPs in rats bearing 9L and RG2 brain tumors. The results demonstrate that the pH_e_ readout was unaffected by the presence of SPIO-NPs, because the intratumoral-peritumoral pH_e_ gradients were essentially identical before and after the infusion of SPIO-NPs, despite slight variations in LWs of the proton peaks for TmDOTP^5−^. The measured pH_e_ was lowest inside the tumor and increased with the distance from the center of mass of the tumor in the more aggressive RG2 tumors. However, in the less aggressive 9L tumors, pH_e_ was notably higher immediately outside the tumor boundary. We envisage coinjection of BIRDS agents (e.g., TmDOTP^5−^) and NPs containing drugs and SPIO, as a new methodology that can deliver high drug payloads to the tumor, image drug distribution, and track tumor location/size (by MRI), and at the same time monitor pH_e_ response to therapy (by BIRDS) [74].

The brain's microvasculature is either degraded or immature in several neuropathologies, including glioblastomas. Breakthroughs in glioma imaging and therapy exploit the fact that NPs, containing either SPIO (for MRI) or drugs (for therapy), can extravasate through the leaky microvasculature. The SPIO-NPs extravasate into the tumor to generate superior MRI contrast while tumor-targeted D-NPs safely deliver high payloads of drugs to the tumor [74].

In the present study, the highest *R*_2_ enhancement (from TmDOTP^5−^ and SPIO-NPs) occurred in the tumor and was lowest in healthy/nontumor tissue farthest from the tumor. Because the *R*_2_ enhancement comes entirely from the infused agents, this region-specific enhancement suggests a corresponding spatial variation in vascular permeability and consequent extravasation. In addition to the enhanced extravasation, the chaotic vascular architecture in tumors contributes to poor clearance leading to increased retention of SPIO-NPs in the interstitial space of the tumor core. By using the *R*_2_ enhancement and the relaxivity of Molday ION (SPIO-NPs), we calculated that the amount of SPIO-NPs in the tumor was 2 to 4 times higher than in healthy/nontumor tissue. The EPR in tumors has been widely utilized to preferentially deliver high amounts of imaging agents and D-NPs, both passively and actively [75, 76].

High-grade solid brain tumors tend to develop necrotic cores due to a combination of poor vascularization and inadequate perfusion [77–79]. Because gliomas like RG2 are very aggressive, they rapidly invade to induce severe neurological problems. As a consequence the rodent reaches terminal situations before the tumor cores are able to become necrotic. For example, these rodent brain tumors grow within a few weeks, whereas in the human brain gliomas develop necrotic foci after many months, if not longer. Tumor necrosis has very likely not yet occurred in these rodent tumors at the time points of our experiments. The observed higher *R*_2_ in the center relative to the periphery suggests higher permeation and accumulation of SPIO-NPs in the center of the tumor due to greater extent of BBB disruption within the tumor niche. Prior studies support these observations. Beaumont et al. did not observe any necrosis in their RG2 rat gliomas at similar time points as our experiments [16]. Their staining results also showed that the BBB was significantly disrupted at the center of the tumor in RG2 tumors. While the vasculature at the primary tumor site/core is leaky, the blood vessels at the tumor infiltration sites (i.e., periphery) are often immature, which may slow the extravasation of SPIO-NPs out of the blood into these new tumor sites. Uehara et al. also showed that necrosis of tumor cores is minimal or absent in RG2 tumors at time points less than 4 weeks following inoculation [80]. Therefore based on the information regarding RG2 tumor growth from prior work in this and other laboratories, we expect the tumor cores to be non-necrotic, and thus higher *R*_2_ increase from the SPIO-NPs would be observed in the tumor core. Additionally, because gliomas including RG2 are known to have an increased presence of macrophages relative to healthy brain tissue, the higher amount of SPIO-NPs in the tumor could be due in part to macrophage phagocytosis [16].

### 4.1. Superparamagnetic Iron Oxide Nanoparticles in Cancer Theranostics

Owing to their strong superparamagnetic properties, tunable size, shape, coating, and magnetic susceptibility, SPIO-NPs have gained utility as therapeutic agents in alternating magnetic field hyperthermia [81–85], as MRI contrast agents for cell tracking [86–89], and for imaging tumor location/size as well as drug delivery [40, 74, 90]. Drug delivery imaging with SPIO-NPs is often accomplished by coencapsulating drugs and SPIO into a given nanocarrier platform (e.g., micelles or liposomes). In liposomes, for example, SPIO-NPs and hydrophilic drugs can be encapsulated inside the nanocarrier, whereas hydrophobic drugs can be incorporated on the nanocarrier membrane. Recent advances also involve coating the surface of SPIO-NPs itself with drugs [11, 12]. Entry and accumulation of these drug-containing and SPIO-containing NPs into the tumor have been achieved by passive targeting, whereby the NPs are small enough to extravasate through leaky tumor vasculature, but large enough not to cross the intact vessels in healthy/nontumor tissue. However, better and more selective targeting is achieved when the NPs are coated with ligands that are specific to receptors and/or transporters that are overexpressed on tumor cells and vasculature. Examples of such targets include transferrin receptors, epidermal growth factor receptors, folate receptors, vascular endothelial growth factor receptors, monocarboxylate transporters, and glucose transporters [29, 91–96]. In all these cases, the delivery and biodistribution of D-NPs are visualized and quantified through signal attenuation (negative contrast) of the *R*_2_-weighted MRI resulting from the strong superparamagnetic fields generated by SPIO-NPs. Because both the drugs and SPIO-NPs are contained in the same nanocarrier, the location and distribution of the SPIO-NPs, as observed by MRI, reflect the biodistribution of D-NPs. By quantifying the SPIO-induced MRI contrast attenuation, it is possible to quantify the D-NPs delivered to the tumor.

Currently, measurement of tumor size is the only FDA-approved method to assess the response to therapy noninvasively. Because changes in tumor size following treatment may take up to a month to manifest, this method is not ideal for aggressive brain cancers, especially when the treatment is later found not to have been effective. Thus a clear need exists for methods that can provide prompt assessment of therapeutic efficacy so that treatment can be altered quickly if desired. Recently, it was shown that quantitative monitoring of the tumor microenvironment following a pharmacologic challenge provides a better way to monitor therapeutic efficacy [97]. Because acidification of pH_e_ promotes drug resistance, degradation of the extracellular matrix, angiogenesis, tumor invasion, and metastasis, drugs that raise (or neutralize) pH_e_ by targeting the acid-generating glycolysis in tumors have demonstrated significant inhibition of tumor growth and enhanced apoptosis [45, 46, 48, 72, 98, 99]. Additionally, drugs that directly raise tumor pH_e_ (e.g., bicarbonate treatment) inhibit tumor invasion and metastasis [100, 101]. Because bicarbonate and drugs that inhibit glycolysis elevate pH_e_ in a few days, methods that quantitatively measure tumor pH_e_ longitudinally may provide an effective evaluation of their therapeutic efficacy and allow for prompt modification of therapy if the initial treatment is not working. A recent study has reported that temozolomide, which is an alkylating agent and is adjuvant chemotherapy used to clinically treat glioblastomas, arrests glioma growth and normalizes intratumoral pH_e_ [102].

### 4.2. Combining Drug Delivery Imaging with pH_e_ Imaging to Assess Therapy

Given the significant relaxation enhancement of the nonexchangeable protons on the TmDOTP^5−^ agent [60, 103, 104] due to pseudocontact interactions with unpaired Tm^3+^ electrons, we hypothesized that BIRDS-based pH_e_ readout of TmDOTP^5−^ will remain uncompromised by SPIO-NPs. Although SPIO-NPs altered MRI contrast in all tissues, SPIO-based MRI contrast clearly demarcated the tumor boundary due to greater extravasation of NPs through leaky blood vessels. Nonetheless, the quality of BIRDS-based pH_e_ readout with TmDOTP^5−^, for both intratumoral and peritumoral regions, was unaffected by the presence of the SPIO-NPs, since the pH_e_ maps obtained before and after the infusion of SPIO-NPs were very similar.

While separate infusions of TmDOTP^5−^ and SPIO-NPs were employed in the present study, future studies might assess the possibility of combining them [74]. Conjugating several monomers of the pH_e_-sensitive agent on the surface of the NPs could possibly enhance the sensitivity of BIRDS to monitor the immediate environment of D-NPs and prolong their lifetime to enable multiple monitoring sessions at various treatment time points. Ordinarily, BIRDS agents have fast renal clearance owing to their small size and thus renal inhibition is necessary for accumulation [57, 58, 60, 105]. However, if conjugated to NPs, the BIRDS agents lifetime might increase significantly (i.e., to several days, which is the case for SPIO-NPs), thus allowing their use without inhibition of renal clearance and obviating the need for repeated infusions [106]. Towards this goal, it has been previously demonstrated in vitro that encapsulation of BIRDS agents in liposomal nanoparticles resulted in an MR signal amplification without impeding the local pH readout [62].

## 5. Summary

The treatment of brain gliomas is hampered in part by a limited availability of reliable in vivo methodologies that can simultaneously and noninvasively measure glioma invasion, drug delivery, and its therapeutic benefits. In this study, we demonstrated superb MRI contrast enhancement and tumor delineation with SPIO-NPs and quantitative imaging of intratumoral-peritumoral pH_e_ gradients using BIRDS in rat models of brain gliomas. Furthermore, we demonstrated that both the intratumoral and peritumoral pH_e_ readouts, measured with BIRDS using TmDOTP^5−^, are not compromised by the presence of SPIO-NPs. Thus, we propose a new cancer imaging protocol that can target high drug payloads (via D-NPs) to tumors and image the drug delivery (via SPIO-NPs), concurrently map tumor location and size (by MRI), and at the same time monitor therapeutic efficacy through drug-induced changes in pH_e_ (by BIRDS) [74].

## Supplementary Material

Figure S1. Prussian Blue Staining for iron (SPIO-NPs) distribution.
Figure S2. Effect of TmDOTP^5-^ and SPIO-NPs infusion on the transverse relaxation rate (R_2_) in Probenecid-infused glioma-bearing animals.
Figure S3. Extracellular pH (pH_e_) and TmDOTP^5-^ linewidths (LW) measured before and after SPIO-NPs infusion in Probenecid-infused glioma-bearing animals.
Figure S4. Comparison of Extracellular pH (pH_e_) in 9L tumor-bearing animals that underwent renal ligation or Probenecid co-infusion to inhibit renal clearance.

## Figures and Tables

**Table 1:** Regional analysis for relaxation rate (*R*_2_) for all RG2 tumor-bearing rats that underwent coinfusion of TmDOTP^5−^ and probenecid (*n* = 5). See Figure 3(a) for details. *R*_2_ was measured inside the MRI-defined tumor core (see Figure S2), at the tumor edge (regions 1 mm outside the tumor boundary), and in the healthy/nontumor tissue before and after the infusion of SPIO-NPs. Data shown are mean and standard deviation (SD).

*R*_2_	Intrinsic		TmDOTP^5−^		TmDOTP^5−^ + SPIO-NPs	
	Mean	SD	Mean	SD	Mean	SD
Tumor core	22.5	2.1	24.7	2.5	32.7	5.2
Tumor's edge	22.7	4.0	23.7	4.4	28.6	7.9
Nontumor tissue	23.8	3.8	23.2	3.4	27.3	3.7

**Table 2:** Regional analysis for extracellular pH (pH_e_) imaging before and after infusion of SPIO-NPs for all RG2 tumor-bearing rats that underwent coinfusion of TmDOTP^5−^ and probenecid (*n* = 5). See Figure 3(b) for details. The pH_e_ was measured inside the MRI-defined tumor core (see Figure S2), at the tumor edge (regions 1 mm outside the tumor boundary), and in the healthy/nontumor tissue before and after the infusion of SPIO-NPs. Data shown are mean and standard deviation (SD).

pH_e_	Before SPIO-NPs		After SPIO-NPs	
	Mean	SD	Mean	SD
Tumor core	6.9	0.1	6.9	0.1
Tumor's edge	6.9	0.1	6.9	0.1
Nontumor tissue	7.2	0.1	7.2	0.1

## Abbreviations

3-APP: 3-Aminopropyl phosphonate
BBB: Blood brain barrier
BIRDS: Biosensor imaging of redundant deviation in shifts
CEST: Chemical exchange saturation transfer
CSI: Chemical shift imaging
EPR: Enhanced permeation and retention
FOV: Field of view
LW: Linewidth
MR: Magnetic resonance
NPs: Nanoparticles
pH_e_: Extracellular pH
ROI: Region of interest
SD: Standard deviation
SNR: Signal-to-noise ratio
SLR: Shinnar-Le Roux
SPIO: Superparamagnetic iron oxide
TmDOTP^5-^: Thulium 1,4,7,10-tetraazacyclododecane-1,4,7,10-tetrakis methylene phosphonate.
