# Supplementary material for: Mapping Extracellular pH of Gliomas in Presence of Superparamagnetic Nanoparticles: Towards Imaging the Distribution of Drug-Containing Nanoparticles and Their Curative Effect on the Tumor Microenvironment
Source: Contrast Media Mol Imaging. 2017 Nov 22;2017:3849373. doi: 10.1155/2017/3849373 (PMC5736903; doi:10.1155/2017/3849373)
Supplement: Supplementary file 1 — Figure S1. Prussian Blue Staining for iron (SPIO-NPs) distribution. Figure S2. Effect of TmDOTP5- and SPIO-NPs infusion on the transverse relaxation rate (R2) in Probenecid-infused glioma-bearing animals. Figure S3. Extracellular pH (pHe) and TmDOTP5- linewidths (LW) measured before and after SPIO-NPs infusion in Probenecid-infused glioma-bearing animals. Figure S4. Comparison of Extracellular pH (pHe) in 9L tumor-bearing animals that underwent renal ligation or Probenecid co-infusion to inhibit renal clearance. [file 3849373.f1.pdf]

Figure S1. Prussian Blue Staining for iron (SPIO-NPs) distribution

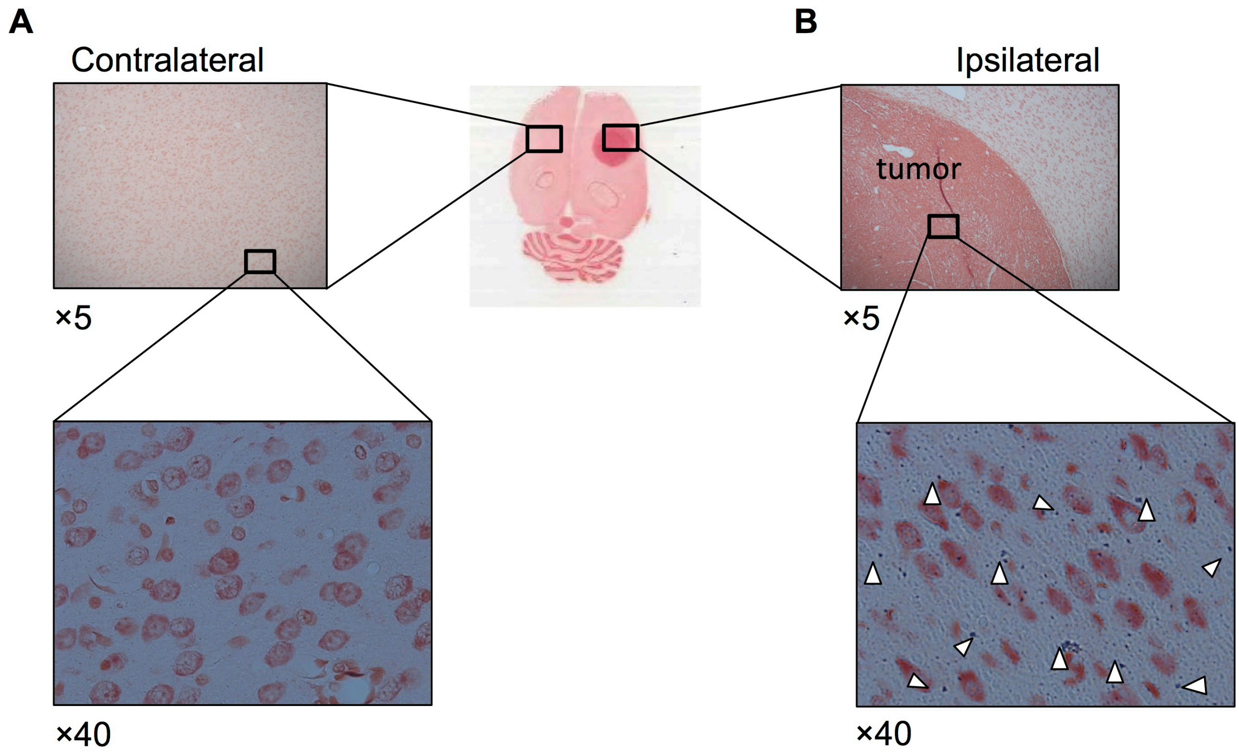

Prussian blue staining for Fe^{3+} in 10 μm thick sections of an RG2 glioma-bearing rat that had undergone renal ligation following infusion of SPIO-NPs. **(A)** SPIO-NPs were not observed in the healthy/non-tumor brain tissue on the contralateral side, consistent with the R_2 results from MRI (**Fig. 1C**). **(B)** However, many clusters of SPIO-NPs were observed inside the tumor (blue dots indicated with white arrows), consistent with the R_2 enhancement observed with MRI after the infusion of SPIO-NPs (**Fig. 1C**). Red color indicates nucleus.

Figure S2. Effect of $TmDOTP^{5-}$ and SPIO-NPs infusion on the transverse relaxation rate (R_2) in Probenecid-infused glioma-bearing animals

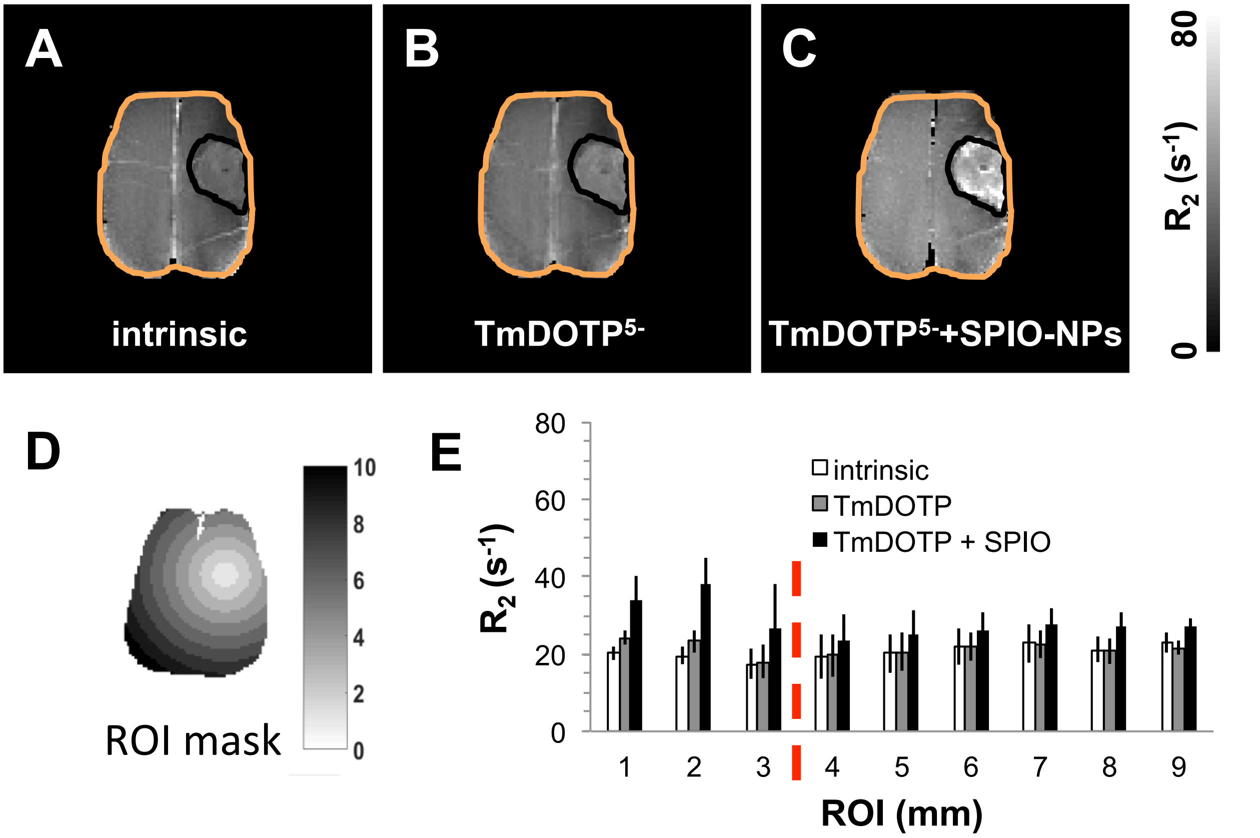

Transverse relaxation rate (R_2) maps of an RG2 glioma-bearing rat that underwent co-infusion of probenecid and $TmDOTP^{5-}$, **(A)** without any contrast agent, **(B)** after infusion of $TmDOTP^{5-}$, and **(C)** after infusion of SPIO-NPs. The scale bar in **A-C** denotes R_2 values from 0 to 80 s^{-1}. Compared to the R_2 map before $TmDOTP^{5-}$ infusion **(A)**, a slight R_2 enhancement was observed throughout the brain after infusion of $TmDOTP^{5-}$ **(B)**, but much greater R_2 enhancement and best tumor delineation was observed following the infusion of SPIO-NPs **(C)**. The contrast enhancement from both $TmDOTP^{5-}$ and $TmDOTP^{5-}$ with SPIO-NPs was region-specific, with a higher enhancement inside the tumor and lower outside of the tumor (relative to the intrinsic contrast, **(A)**). The black outline in **A-C** denotes the tumor boundary, which is based on the MRI contrast after the infusion of SPIO-NPs. The region of interest (ROI) mask based on 1 mm circular rings from the tumor center **(D)** was used to generate the radial R_2 distribution histogram **(E)**. The scale bar in **D** denotes 0 to 10 mm diameter circular ROIs (portrayed on a representative rat brain slice). The average R_2 values inside the tumor were 19.1, 21.8 and 32.9 s^{-1} before contrast agent infusion, after infusion of $TmDOTP^{5-}$, and after infusion of SPIO-NPs, respectively. For the healthy tissue (contralateral side), the average R_2 values were 21.7, 21.4 and 26.9 s^{-1} before contrast agent administration, after infusion of $TmDOTP^{5-}$, and after infusion of SPIO-NPs, respectively. The gray dashed line in **E** denotes the demarcation between tumor and non-tumor regions. Based on the in vitro relaxivity of Molday ION, the amount of SPIO-NPs in the tumor was 2.1 times greater than in healthy tissue suggesting a preferential extravasation and accumulation in the tumor. See Fig. 1 for an example of R_2 maps of an RG2 glioma-bearing rat that underwent renal ligation for $TmDOTP^{5-}$ infusion.

Figure S3. Extracellular pH (pH_e) and $TmDOTP^{5-}$ linewidths (LW) measured before and after SPIO-NPs infusion in Probenecid-infused glioma-bearing animals

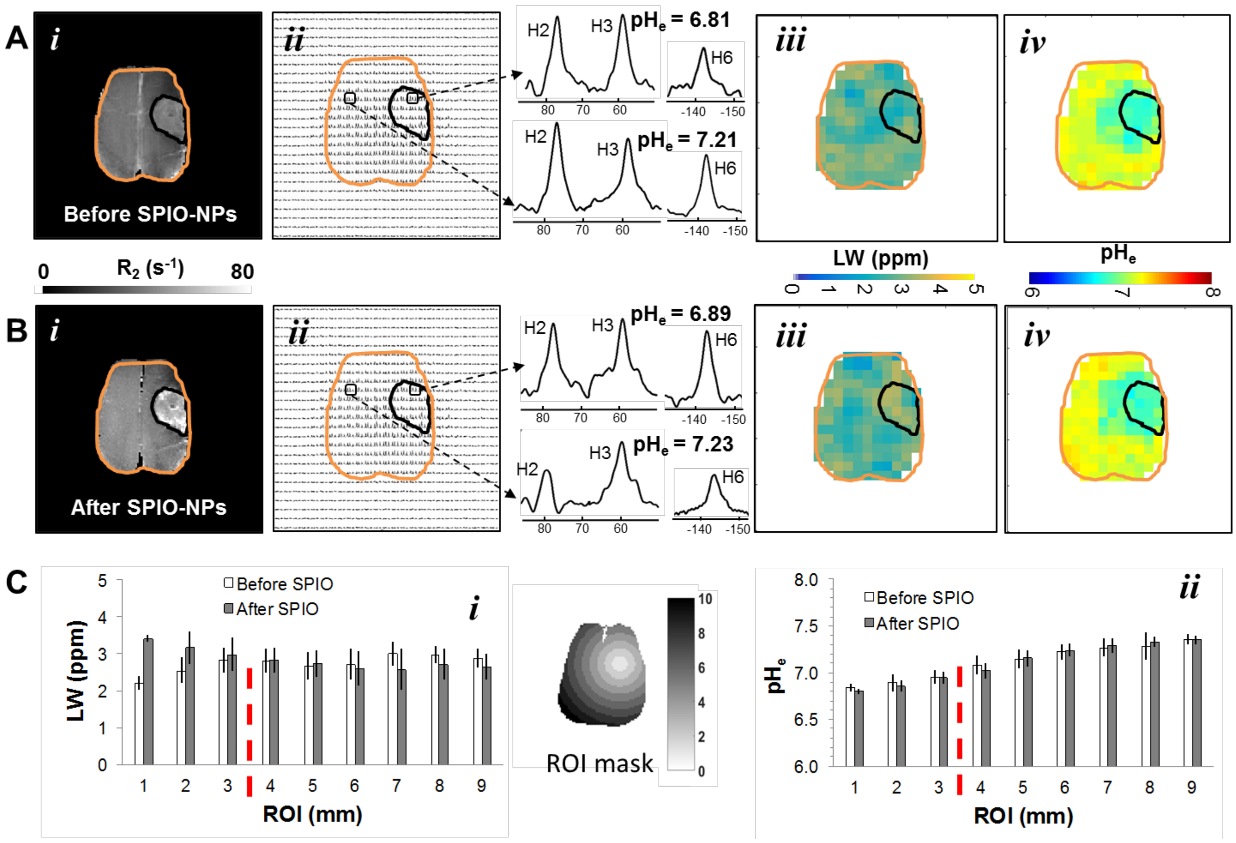

Multi-modal data of relaxation rate (R_2) maps, chemical shift imaging (CSI) maps, linewidth (LW), and extracellular pH (pH_e) maps obtained for the same RG2 tumor-bearing rat, which had undergone co-infusion of $TmDOTP^{5-}$ and probenecid. **(A*i*-*iv*)** represents maps before the SPIO-NPs infusion while **(B*i*-*iv*)** represents the maps after the infusion. The R_2 maps were used to delineate and localize the tumor (black outline) and brain (orange outline) boundaries on the CSI, LW, and pH_e maps. R_2 values inside the tumor increased significantly after infusion of SPIO-NPs. The CSI maps were used create the LW maps (based on the H6 peak of $TmDOTP^{5-}$) and pH_e maps. The LW increased after infusion of SPIO-NPs especially in the tumor. The pH_e within the tumor core and on the tumor margin was lower than healthy/non-tumor regions. The panels between the CSI and the LW maps show examples of ^1H spectra of $TmDOTP^{5-}$ protons from voxels inside and outside the tumor, revealing a significant intratumoral and peritumoral LW and pH_e difference. A more detailed comparison was done using a region of interest (ROI) analysis of LW **(C*i*)** and pH_e **(C*ii*)** maps before and after the infusion of SPIO-NPs, using the mask shown. The scale bar in the mask denotes 0 to 10 mm diameter circular ROIs (portrayed on a representative rat brain slice). The red dashed line denotes the demarcation between tumor and non-tumor regions. The average pH_e before infusion of SPIO-NPs was 6.85 ± 0.03 within the tumor, and pH 7.15 ± 0.06 for healthy tissue (**A*iv***). The pH_e measured after infusion of SPIO-NPs was pH 6.86± 0.07 within the tumor and pH 7.17 ± 0.06 in the healthy tissue **(B*iv*)**. The pH_e of the tumor edge (ROI 4) was also relatively acidified (pH 6.98 ± 0.13 before and 6.90 ± 0.09 after infusion of SPIO-NPs) compared to healthy tissue farthest from the tumor core (ROIs 5-9). See **Fig. 2** for an example of multi-modal data of an RG2 tumor rat that underwent renal ligation.

Figure S4. Comparison of Extracellular pH (pH_e) in 9L tumor-bearing animals that underwent renal ligation or Probenecid co-infusion to inhibit renal clearance

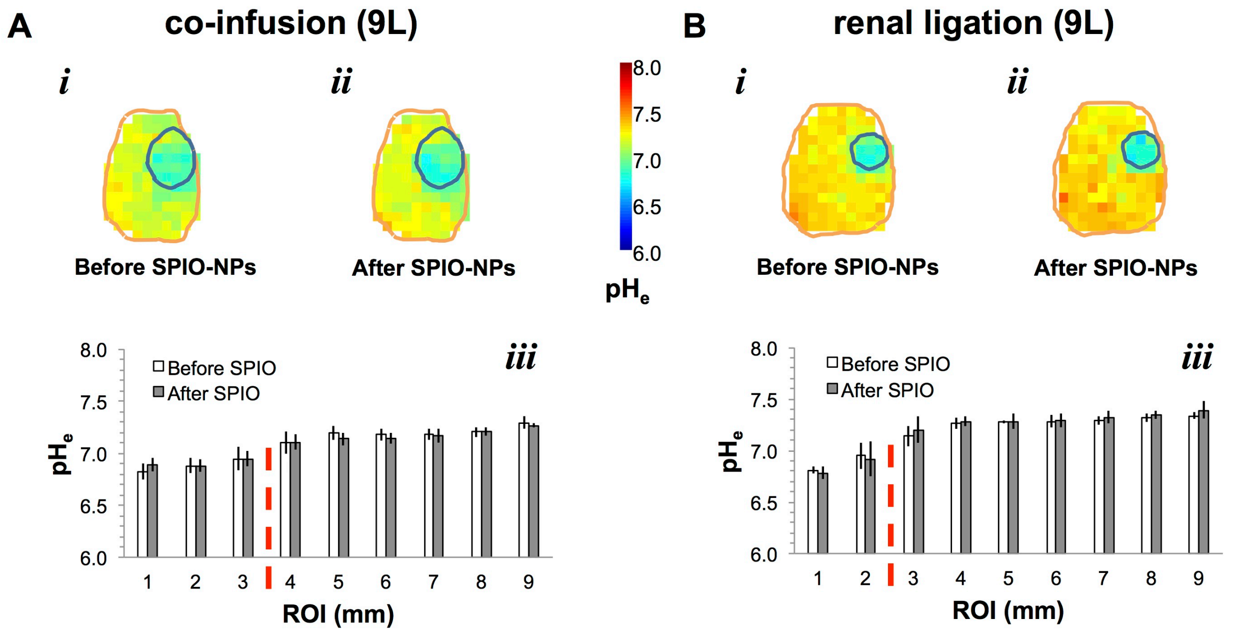

Comparison of pH_e maps for 9L tumors before and after infusion of SPIO-NPs in rats that underwent **(A)** co-infusion of $TmDOTP^{5-}$ and probenecid and **(B)** infusion of $TmDOTP^{5-}$ after renal ligation. In both **A** and **B**, **(*i*)** and **(*ii*)** represents the pH_e maps before and after SPIO-NPs infusion, respectively, while **(*iii*)** depicts a detailed ROI analysis. See **Fig. 1** for details of the ROI mask. In both **A** and **B**, the pH_e inside the tumor was lower (acidified) compared to healthy/non-tumor tissue, but the acidification was restricted to the MRI-defined tumor core only. See **Fig. 4** for a comparison of regional pH_e dependence in RG2 versus 9L tumors in rats that underwent co-infusion of $TmDOTP^{5-}$ and probenecid.
